# Adaptation and Validation of the Authoritative School Climate Survey in a Sample of Chilean Adolescents

**DOI:** 10.3389/fpsyg.2021.573457

**Published:** 2021-02-12

**Authors:** José Luis Gálvez-Nieto, Francisco Paredes, Italo Trizano-Hermosilla, Karina Polanco-Levican, Julio Tereucán-Angulo

**Affiliations:** ^1^Departament of Social Work, University of La Frontera, Temuco, Chile; ^2^Department of Psychology, University of La Frontera, Temuco, Chile; ^3^Department of Psychology, Temuco Catholic University, Temuco, Chile

**Keywords:** authoritative school climate, adolescence, school norms, institutional authority, reliability, validity

## Abstract

Authoritative school climate is a relevant and novel construct that improves the academic performance and social-emotional development of students. This study aimed to evaluate the psychometric properties of reliability and validity of the Authoritative School Climate Survey (ASCS) in a sample of Chilean adolescents. A cross-sectional study was carried out, in which 808 students from 12 schools in Chile participated (55.1% men and 44.9% women), with a mean age of 15.94 (*SD* = 1.32). The results obtained through exploratory and confirmatory factor analyzes ratified the hypothesized structure of two correlated factors. As expected, evidence of criterion validity showed significant relationships between the measures of authoritative school climate and attitude toward institutional authority. This study provides evidence regarding the psychometric quality of the scale to assess authoritative school climate, allowing its use in the Chilean context.

## Introduction

School climate has been abundantly defined in the international literature, which has generated conceptual and measurement difficulties (Thapa et al., 2013; Wang and Degol, 2016; Rudasill et al., 2018). However, there is relative agreement that school climate is a multidimensional construct (Thapa et al., 2013) that refers to the quality and character of school life (Cohen et al., 2009), describing it as the heart and soul of the school (Freiberg and Stein, 1999). One of the main dimensions of school climate is the academic relationship between teachers and students, with various studies suggesting that students who perceive high levels of teacher support present academic success (Crowley et al., 2019; Alonso-Tapia et al., 2020).

School climate has been associated with a variety of positive outcomes which go beyond academic achievement (Li et al., 2020; Trinidad, 2020; Zysberg and Schwabsky, 2020). A recent study conducted with four measures of school climate reported by students (*n* = 823,753), concluded that a positive school climate promotes student attendance (Hamlin, 2020). Another recent study (Daily et al., 2020) analyzed the longitudinal association between school climate and the initiation of substance use, concluding that a positive school climate can delay the initiation of substance use and promote academic success. Additionally, school climate has been associated with the development of self-concept and self-esteem (Coelho et al., 2020) and favors prosocial behavior (Villardón-Gallego et al., 2018).

On the other hand, educational establishments with deteriorated school climates exhibit higher levels of bullying (Cui and To, 2020), problems with respect for authority (Gálvez-Nieto et al., 2020a) and behavioral problems (Jiménez Gutiérrez and Lehalle, 2012). An interesting study analyzed patterns of dating aggression, victimization and school environment in a sample of 4,114 adolescents, with the results indicting that deteriorated school climate was associated with aggression and victimization in dating (Sullivan et al., 2020). A study applied to 2,560 schools in the United States (Sulak, 2018), established that deteriorated school climate is explained by structural factors such as geographic location in high-crime sectors and large campuses serving over 1,000 students.

Additionally, international evidence suggests that a positive school climate characterized by high expectations and favorable relationships with teachers is associated with higher academic performance (Barile et al., 2012; Brault et al., 2014), adequate social relationships between students (Steffgen et al., 2013) and a lower probability of being a victim of aggression (Elsaesser et al., 2013).

### Theoretical Background

The theory of ecological systems (Bronfenbrenner, 2002) is one of the most frequently used theories to interpret the school climate (Wang and Degol, 2016). This theory states that human development is a joint function between people and their social context. According to this theory, the different social subsystems directly and indirectly influence individual behaviors.

Applied to the school context, the behavior of individuals is influenced by multiple variables located in the different social and school subsystems (Rudasill et al., 2018). The first ecological level is the microsystem. In this subsystem, the direct relationships between the members of the educational community are evident and give character and tone to the school climate. The mesosystem is a function of the interactions between the members of the educational community and represents a space where highly relevant variables of the school climate are located. The exosystem is composed of a series of factors that must be considered as potential influences on the school climate. On the other hand, the macrosystem is composed of all structures where the student does not participate directly, such as values or culture. Finally, the chronosystem represents a broader level based on historical events and movements that influence the beliefs, priorities and norms of the culture.

### Authoritative School Climate: Conceptual Definition and Its Relationship With Respect for Authority Figures

The authoritative school climate is made up of two dimensions, Disciplinary Structure and Student Support (Gregory and Cornell, 2009; Gregory et al., 2010; Konold et al., 2014; Cornell and Huang, 2016). According to these studies, the first dimension refers to the strict but fair application of school rules, and the Student Support dimension refers to the degree to which students perceive that their teachers are understanding, respectful and willing to help them.

An interesting body of research supports the authoritative school climate model (Gregory et al., 2010; Konold et al., 2014). For example, the Cornell and Huang (2016) study, involving 47,888 high school students, found that schools with strict but fair disciplinary strategies and supportive teacher-student relationships showed decreased likelihood of student alcohol consumption, marijuana use and bullying, among other variables. Another cross-sectional study of 48,027 high school students (Konold and Cornell, 2015), showed that authoritative school climate was associated with greater school commitment and less peer aggression at both the student and school level, controlling for the demographic effects of the school.

As it has been noted, student attitudes toward teachers as authority figures is associated with respect for legal frameworks inside and outside of educational establishments (Cava et al., 2013). Attitudes of respect for institutional authority have been negatively related to school violence (Cava et al., 2013) and positively related to school climate (Moreno et al., 2009).

### Measurement of School Climate

It has been difficult to reach a consensus regarding the measurement of the school climate construct for various reasons. As has been shown, there is a significant range of definitions (Thapa et al., 2013; Rudasill et al., 2018) due to the great variety of theorized dimensions (Wang and Degol, 2016), the unit of analysis selected for measurement (Maxwell et al., 2017; Grazia and Molinari, 2020), and the use of subjective or objective measurement strategies (Cohen et al., 2009; Wang and Degol, 2016).

Recent systematic review studies (Grazia and Molinari, 2020; Lewno-Dumdie et al., 2020; Marraccini et al., 2020) have provided empirical evidence to select the most appropriate instruments. For example, the study by Marraccini et al. (2020), identified 26 instruments for measuring school climate and concluded that the measures of school climate identified came from a variety of theoretical frameworks, had different dimensions and varied significantly in applicability. Likewise, in the review study carried out by Grazia and Molinari (2020), most of the validated school climate scales were only used in one study, revealing a fragmented field of study that offers low comparability of results.

In Chile, despite the existence of psychometric studies of instruments to assess school climate, research has not yet been conducted to study the specific measure of authoritative school climate. One of the scales with the greatest accumulated psychometric evidence is the Questionnaire to Evaluate the Social Climate of the School. This instrument, originally created in Spain (Trianes et al., 2006), is made up of 14 items and 2 factors: School Social Climate and Teacher Social Climate. In Chile, two recent psychometric studies have been presented (Gálvez-Nieto et al., 2015b, 2017), ratifying the structure of the two correlated factors found in previous studies. Despite empirical evidence, the construct of school social climate and its two dimensions are insufficient to theoretically cover the multidimensionality of school climate (Thapa et al., 2013; Wang and Degol, 2016).

Another scale that a psychometric study in Chile has presented is the School Climate Scale (López et al., 2014). This instrument was originally designed in Israel (Benbenishty and Astor, 2005), derived from an adaptation of the California School Climate and Safety Survey (Furlong, 1996). In another line of research (López et al., 2018), the Classroom Climate Scale, an instrument to measure the specific construct of class climate in primary school students was created and validated in Chile. This instrument presented adequate levels of reliability and validity in the Chilean context and included the dimensions: physical environment, teacher—student interactions, peer relationships, and teacher orientation to learning. In a recent study, the psychometric properties of the Dual School Climate and School Identification Measure-Student (SCASIM-St) were analyzed. SCASIM-St is a double-measure scale that assesses school climate and school identification (Lee et al., 2017) and presented a good psychometric fit in the Chilean context (Gálvez-Nieto et al., 2020b).

To understand the Chilean educational context, it is important to point out that families can select the school that their children will attend. This model is based on competition for higher student enrollment (Ruiz-Tagle, 2019). The schools are classified according to the type of administration that funds them. Public establishments receive financial subsidies from the state and account for 35.5% of the total enrollment in the country. Subsidized private schools receive a mixed contribution from the state and private entities and account for 53.8% of national enrollment. Nonsubsidized private schools, which do not receive state financial subsidies, represent 9.1% of the national enrollment. Finally, 1.6% of enrollment is administered by local education services, which function based on state financial contributions (Ministerio de Educación de Chile, 2020).

Considering the aforementioned information and the relevance of studying school climate using the authoritative theoretical model (Gregory and Cornell, 2009; Gregory et al., 2010; Konold et al., 2014; Cornell and Huang, 2016), we conducted a study of psychometric evaluation. The hypotheses of this study are the following: (1) the scores of the Authoritative School Climate Survey (ASCS) will have a factorial structure of two factors, Disciplinary Structure and Student Support, in addition to adequate levels of reliability; and (2) the scores of the scale of the ASCS will present significant correlations with the Attitudes to Institutional Authority in Adolescence Scale (AIA-A). Therefore, this study aims to evaluate the psychometric properties of reliability and validity of the authoritative school climate scale in a sample of Chilean adolescents.

## Method

### Participants

The study was conducted in the Los Lagos Region of Chile with 808 adolescent student participants. The participants were selected from a non-probabilistic sampling in 12 schools: public schools (32.08%) and subsidized private schools (69.92%). The sample was made up of students of both genders (55.1% men and 44.9% women). The average age of the adolescents was 15.94 years (*SD* = 1.32). Regarding the family structure of the students, 50.5% lived with both parents, 35.8% lived only with the mother, 8.4% with other relatives, 4.7% only with the father, and 0.6% with guardians. The students’ families lived mainly in urban areas (82.1%). The schools that agreed to participate in the study were made up of students from various socioeconomic levels, but mainly represented low and middle levels (Table 1).

**TABLE 1:** Description of the sample.

**Variable**	**Total sample**	**%**	**Exploratory sample**	**%**	**Confirmatory sample**	**%**
**Ethnic origin**						
Yes	225	27.8	104	25.7	121	30
No	583	72.2	300	74.3	283	70
**Grade level**						
1°	90	11.1	40	9.9	50	12.4
2°	130	16.1	75	18.6	55	13.6
3°	511	63.2	253	62.6	258	63.9
4°	77	9.5	36	8.9	41	10.1
**Area of residence**						
Urban	663	82.1	327	80.9	336	83.2
Rural	145	17.9	77	19.1	68	16.8

To provide evidence of cross-validity, 2 independent sub-samples of 404 students each were randomly selected (Table 1). To ensure the equivalence of both samples, percentage differences between men and women were estimated [χ^2^(*df* = 1) = 1.271; *p* = 0.260], ethnic origin [χ^2^(*df* = 1) = 1.280; *p* = 0.182], grade level [χ^2^(*df* = 3) = 4.562; *p* = 0.207], area of residence [χ^2^(*df* = 1) = 1.780; *p* = 0.182], in addition to estimating the difference between the average ages [*t*(*df* = 805) = 0.398; *p* = 0.691].

### Instruments

To achieve the research objectives, the students answered three instruments simultaneously. The first was a sociodemographic questionnaire that included the variables age, gender, grade, and area of residence.

The second instrument was the ASCS, a self-report scale originally developed in the United States to assess the authoritative school climate (Gregory and Cornell, 2009; Gregory et al., 2010; Konold et al., 2014; Konold and Cornell, 2015; Cornell and Huang, 2016). The ASCS is made up of 15 items, of these, 13 items are formulated in a direct way and two items (item 4 and item 5) in a reversed way (Vigil-Colet et al., 2020). The items were answered using a 5-point scale (1 = never, 2 = a few times, 3 = sometimes, 4 = frequently, 5 = always). The ASCS has two factors: Disciplinary Structure, which consists of seven items that measure the degree of impartiality of school discipline (items 1–7, e.g., “Students are treated fairly regardless of their race or ethnicity”); and Student Support, which consists of eight items that assess the perception of support provided by the teachers and professional staff of the school (items 8–15, e.g., “If I tell a teacher that someone is bullying me, the teacher will do something to help”). Evidence of reliability and validity in the United States (Gregory and Cornell, 2009; Gregory et al., 2010; Konold et al., 2014; Cornell and Huang, 2016) showed adequate psychometric adjustment indicators in terms of factor structure and reliability.

To assess the evidence of convergent validity, the AIA-A was applied. The AIA-A is a self-report scale that measures the attitudes of adolescents toward institutional authority (Cava et al., 2013). The items were answered using a 5-point scale (1 = never, 2 = a few times, 3 = sometimes, 4 = frequently, 5 = always). The factorial structure of the AIA-A is made up of two factors: Positive Attitude to Authority (5 items, e.g., “The police are there to make a better society for all”), referring to the degree of respect toward teachers and the police; and Positive Attitude to Transgression (4 items, e.g., “It is normal to break the law if no one is harmed”), referring to positive attitudes toward transgression of school rules. Evidence of reliability and validity in Chile (Gálvez-Nieto et al., 2015a), and simultaneously in Chile and Colombia (Gálvez-Nieto et al., 2018) showed adequate psychometric adjustment indicators in terms of factor structure and reliability.

### Process

For the linguistic adaptation of the instrument, the criteria established by Muñiz et al. (2013) was followed. First, the scale was translated from English to Spanish using the inverse translation method. Subsequently, the instrument was presented to an expert committee which evaluated the items qualitatively in terms of their cultural relevance in the Chilean context. Finally, a pilot test of the instrument was carried out, providing a preliminary evaluation of the quality of the linguistic adaptation process.

For the administration of the instruments, contact was made with the school directors and a request was made to sign a work agreement with the research team. Next, informed consents were sent to the parents of the students. Once the parent authorizations were obtained, an informed consent was applied to the students participating in the study. After the ethical principles of the project were verified, the instruments were administered during the first hour of class.

### Data Analysis

First, descriptive analyses were carried out for the ASCS items. Later, an exploratory factor analysis (EFA) was performed with the first sample using the polychoric correlation matrix and the unweighted least squares estimation method (ULS). Assuming that the items were correlated, an oblique rotation was implemented. To decide the number of factors, we used Minimum Rank Factor Analysis procedure (Ten Berge and Kiers, 1991) was used. These analyzes were performed with FACTOR 10.8.03 (Lorenzo-Seva and Ferrando, 2006). The item selection criteria were as follows: saturations greater than or equal to 0.30 in the theoretically defined factor, and absence of cross saturations greater than 0.30 in both factors.

A confirmatory factor analysis (CFA) was performed with the second sample using the MPLUS 7.11 software (Muthén and Muthén, 2012). The polychoric correlation matrix was used, as well as the estimation method weighted least squares with mean and variance adjusted (WLSMV; Muthén and Muthén, 2012). The goodness of fit indices used to assess the quality of the model included the comparative fit index (CFI), Tucker-Lewis index (TLI), and root mean square error of approximation (RMSEA). For the former indices (CFI and TLI), values greater than 0.90 were considered an acceptable fit for the model (Schumacher and Lomax, 1996), and values less than 0.08 a reasonable fit (Browne and Cudeck, 1993). Cronbach’s alpha coefficient was used to evaluate reliability. Additionally, given the limitations of this estimator (Green and Yang, 2015; Trizano-Hermosilla and Alvarado, 2016), McDonald’s omega coefficient was also used.

## Results

### Descriptive Analyses

Table 2 shows the means, standard deviation, skewness and kurtosis of the 15 items in the scale. The descriptive analysis for the mean of the items yielded a maximum mean of 4.19 (*SD* = 1.028), which corresponded to item 3 “Students are treated fairly regardless of their race or ethnicity.” The minimum mean (*M* = 3.08, *SD* = 0.784) corresponded to item 5 “The adults at this school are too strict.”

**TABLE 2:** Descriptive statistics.

**Ítems**	**Mean**	**Std. Deviation**	**Skewness**	**Kurtosis**	**EFA 1**		**EFA 2**		**EFA 3**		**EFA 4**	
					**F1**	**F2**	**F1**	**F2**	**F1**	**F2**	**F1**	**F2**
It1 The punishment for breaking school rules is the same for all students/El castigo por no respetar las reglas del colegio es el mismo para todos los estudiantes	3.56	1.119	–0.224	–0.825	0.083	**0.521**	**0.457**	0.095	0.093	**0.481**	0.095	**0.494**
It2 Students at this school only get punished when they deserve it/Los estudiantes del colegio solamente son castigados cuando se lo merecen	3.58	1.088	–0.330	–0.635	−0.076	**0.714**	**0.636**	−0.075	−0.096	**0.696**	−0.098	**0.704**
It3 Students are treated fairly regardless of their race or ethnicity/Los estudiantes son tratados de manera justa, independientemente de su origen étnico o de su cultura	4.19	1.028	–1.280	1.118	0.187	**0.530**	**0.482**	0.184	0.188	**0.505**	0.187	**0.515**
It4 Students get suspended without good reason/Los estudiantes frecuentemente son suspendidos sin una buena razón	4.05	1.027	–1.144	1.009	0.079	**0.302**	**0.503**	−0.120	−0.051	**0.430**	−0.072	**0.428**
It5 The adults at this school are too strict/Las personas que trabajan en este colegio son muy estrictas	3.08	0.784	0.254	0.456	0.199	−0.297	Removed					
It6 The school rules are fair/Las reglas del colegio son justas	3.56	1.048	–0.225	–0.699	0.310	**0.423**	**0.615**	0.118	0.182	**0.551**	0.166	**0.554**
It7 When students are accused of doing something wrong they get a chance to explain it/Cuando los estudiantes cometemos alguna falta, tenemos la posibilidad de explicarlo	3.61	1.163	–0.445	–0.683	0.542	0.072	0.259	0.382	**0.453**	0.184	Removed	
It8 Most teachers and other adults at this school care about all students/La mayoría de los profesores y personal que trabaja en el colegio se preocupan de todos los estudiantes	3.69	0.956	–0.381	–0.510	**0.594**	0.158	0.292	**0.475**	**0.539**	0.215	**0.512**	0.238
It9 Most teachers and other adults at this school want all students to do well/La mayoría de los profesores y personal que trabaja en el colegio quieren que a los estudiantes les vaya bien	4.10	0.901	–0.922	0.559	**0.674**	0.017	0.161	**0.559**	**0.596**	0.104	**0.582**	0.124
It10 Most teachers and other adults at this school listen to what students have to say/La mayoría de los profesores y personal que trabaja en el colegio escuchan lo que los estudiantes tienen que decir	3.59	0.974	–0.236	–0.627	**0.712**	−0.027	0.161	**0.559**	**0.618**	0.076	**0.570**	0.103
It11 Most teachers and other adults at this school treat students with respect/Los profesores y personal que trabaja en el colegio tratan a los estudiantes con respeto	3.98	0.918	–0.746	0.179	**0.596**	0.155	0.357	**0.418**	Removed			
It12 There are adults at this school I could talk with if I had a personal problem/Hay profesionales o personas en este colegio con quienes yo podría hablar si tuviera un problema personal	3.52	1.326	–0.433	–1.014	**0.635**	−0.045	−0.050	**0.663**	**0.658**	−0.052	**0.654**	−0.039
It13 If I tell a teacher that someone is bullying me, the teacher will do something to help/Si le cuento a algún profesor(a) que alguien me está haciendo bullying este hará algo para ayudarme	4.00	1.044	–0.869	0.067	**0.774**	−0.069	−0.003	**0.743**	**0.765**	−0.035	**0.765**	−0.021
It14 I am comfortable asking my teachers for help with my schoolwork/Me siento cómodo pidiendo ayuda a mis profesores para poder hacer mis tareas o trabajos del colegio	3.65	1.116	–0.546	–0.418	**0.559**	−0.044	−0.129	**0.660**	**0.634**	−0.097	**0.641**	−0.090
It15 There is at least one teacher or another adult at this school who really wants me to do well/En este colegio/liceo, hay al menos un profesor(a) que realmente quiere que me vaya bien	4.12	1.031	–1.120	0.665	**0.589**	0.001	−0.047	**0.653**	**0.625**	−0.025	**0.633**	−0.017

*Matrices of factor loadings. Values in bold highlight the highest factor loadings.*

### Construct Validity Evidence

As no psychometric analyzes of the ASCS had been conducted in Chile, we decided to carry out an EFA with the 15 items on the scale. First, the adequacy of the correlation matrix was evaluated, based on the sample adequacy test (KMO = 0.889) and Bartlett’s χ^2^ test (*df* = 105) = 1469.5; *p* < 0.001, for which both results endorsed the feasibility of performing the EFA. The results of the ULS procedure and the direct oblique rotation showed the presence of two latent factors that explained 45.9% of the total data variance. However, as shown in Table 2, the factor loadings presented results that differed from the original theoretical model. The Disciplinary Structure factor item 5 “The adults at this school are too strict” presented a load of less than 0.30 and was considered insufficient to be included within this factor. Therefore, it was decided to eliminate the item. The second EFA was then performed [KMO = 0.895; Bartlett χ^2^(*df* = 91) = 1438.6; *p* < 0.001], and item 11 “Most teachers and other adults at this school treat students with respect” was eliminated, since it presented cross-loads in both the Disciplinary Structure factor and the Student Support factor. A third EFA [KMO = 0.879; Bartlett χ^2^(*df* = 78) = 1246.6; *p* < 0.001], led to the removal of item 7 “When students are accused of doing something wrong, they get a chance to explain it,” for not presenting factor loadings in the theoretically expected dimension.

Finally, a fourth EFA was conducted [KMO = 0.871; Bartlett χ^2^(*df* = 66) = 1097.1; *p* < 0.001]. The extraction analysis based on the eigenvalue and the parallel analysis suggested maintaining two factors that explained more variance than that expected by random matrices. These factors explained 49.9% of the total variance and corresponded to the original theoretical structure (Cornell and Huang, 2016). The first factor was made up of items 1, 2, 3, 4, and 6, and corresponds to Disciplinary Structure. The second factor, Student Support, grouped items 8, 9, 10, 12, 13, 14, and 15 (Table 2). Both factors presented a moderate, positive and significant correlation (*r* = 0.631; *p* < 0.001).

**FIGURE 1:**
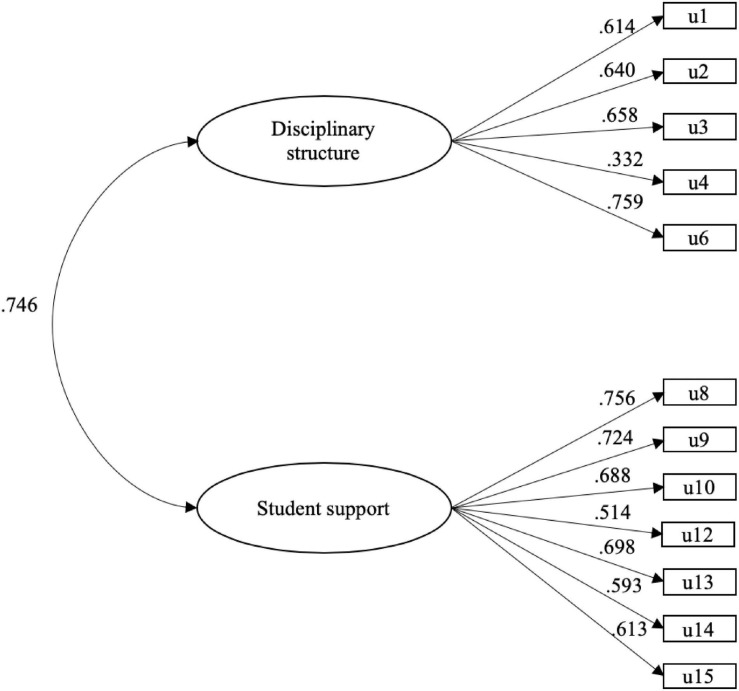
Model of two correlated factors (*n* = 404). All the estimated parameters were statistically significant (*p* < 0.001).

To confirm the structure obtained, a CFA was performed with the data from the second sample. For the two correlated factors model, the goodness of fit indices presented satisfactory *WLSMV-*χ^2^(*df* = 53) = 186.531, *p* < 0.001; CFI = 0.949; TLI = 0.937; RMSEA = 0.054 (CI90% = 0.051–0.057). These values generally indicate that the model fits the data well, thus confirming the proposed two-factor theoretical structure for the authoritative school climate construct (Figure 1).

### Evidence of Criterion Validity

**FIGURE 2:**
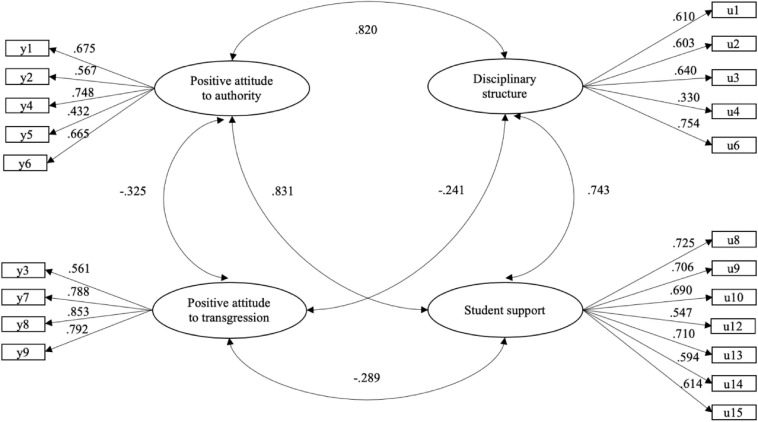
Model of relationships between Authoritative School Climate Survey (ASCS) and Attitudes to Institutional Authority in Adolescence Scale (AIA-A) (*n* = 404). All the estimated parameters were statistically significant (*p* < 0.001).

To evaluate evidence of criterion validity, a structural equation model related the factors of AIA-A and the factors of the ASCS (Figure 2). The result showed satisfactory adjustment indices *WLSMV* -χ^2^(*df* = 183) = 620.472, CFI = 0.956, TLI = 0.949, RMSEA = 0.054 (0.050–0.059), presenting significant, positive, and high correlations between Positive Attitude to Authority and the dimensions Disciplinary Structure (0.820) and Student Support (0.831). Meanwhile, significant, negative and moderate correlations were seen between Positive Attitude to Transgression, Disciplinary Structure (−0.241), and Student Support (−0.289).

### Evidence of Reliability

Regarding the evidence of reliability, all the estimators were satisfactory. The factors with the greatest reliability were Student Support, with a Cronbach’s alpha coefficient of 0.793 (Omega = 0.838); and Disciplinary Structure, with a Cronbach’s alpha coefficient of 0.664 (Omega = 0.725).

## Discussion

This research aimed to evaluate the psychometric properties of reliability and validity of the ASCS in a sample of Chilean adolescents. To achieve this objective, two hypotheses were formulated. The first hypothesis stated that the scores of the authoritative school climate scale would have a factorial structure of two factors called Disciplinary Structure and Student Support, in addition to adequate levels of reliability.

The findings of this study present evidence that support this hypothesis, confirming the presence of a theoretical structure consistent with previous studies (Gregory and Cornell, 2009; Gregory et al., 2010; Konold et al., 2014; Cornell and Huang, 2016). However, as previously stated, three items were eliminated since they did not meet the retention criteria for the final version of the scale, resulting in five items for the Disciplinary Structure factor and seven for the Student Support factor, both factors with adequate levels of reliability. The elimination of these three items can be explained by comprehension problems or the presence of inverse items, which could have affected the factorial structure or reduced model fit (Danner et al., 2015; Vigil-Colet et al., 2020). On the other hand, it is pertinent to specify some characteristics of the educational and cultural context of Chile, for example, the high social stratification of the educational system (Ruiz-Tagle, 2019) and the existence of a significant percentage (12.8%) of people belonging to ethnic groups (Instituto Nacional de Estadísticas, 2018).

The second hypothesis stated that the ASCS scores would present significant correlations with the AIA-A. The findings allowed us to strongly support this hypothesis by confirming positive relationships between Positive Attitude to Authority and the factors of Disciplinary Structure and Student Support, in addition to negative relationships between the Positive Attitude to Transgression and the ASCS factors. These results are consistent with empirical research that suggests that students who perceive positive school climates and relationships between teachers and students tend to present low levels of transgression toward authority (Moreno et al., 2009; Gálvez-Nieto et al., 2018, 2020a). Furthermore, a positive attitude toward rules not only reduces children’s participation in violent behaviors, but also favors academic success (Trinidad, 2020) and their psychosocial adjustment in other social contexts (Bonilla et al., 2017).

In relation to the theoretical and conceptual contributions of this study, there is currently a large variety of conceptualizations of school climate, leading to a dispersion of conceptual approaches that results in empirical findings that are not very consistent and sometimes contradictory (Rudasill et al., 2018; Marraccini et al., 2020). Furthermore, as stated by Grazia and Molinari (2020), most of the school climate scales validated in various contexts have only been used in one study, showing that the field of school climate study is highly fragmented and offers low comparability of results. In contrast, this psychometric study contributes to the comparability of results between cultural contexts.

Regarding the contributions to the Chilean educational practice, the authoritative school climate scale offers a novel measure of school climate which will facilitate decision-making within educational communities. In this sense, the ASCS is a tool that will both stimulate research and help professionals the field of education, especially since it provides a simple way to evaluate the authoritative school climate. Additionally, school climate is a factor that can be improved (Sulak, 2018), meaning that professionals can make efforts to develop the dimensions of Disciplinary Structure and Student Support to promote the well-being and educational success of students (Crowley et al., 2019; Alonso-Tapia et al., 2020).

One limitation of this study is the sample, which despite being large, should be probabilistic and representative of the population. It is necessary to point out that students from non-subsidized private schools did not participate in the study. This aspect is relevant, given that despite representing less than 10% of the total enrollment in Chile (Ministerio de Educación de Chile, 2020), they have demonstrated a climate of respectful relationships with teachers and present lower levels of rule transgression (Gálvez-Nieto et al., 2020a). Another limitation is the age range; this study only includes adolescent students, an aspect that restricts its use to one type of population. Future lines of research should include a probabilistic and representative sample, which would allow the heterogeneity of the different types of educational establishments in Chile to be documented. Additionally, they should contribute evidence of metric invariance between different population subgroups based on gender, indigenous population, migrant population, and expand the age range to encompass elementary school students, among other factors (Gálvez-Nieto et al., 2018).

## Data Availability Statement

The datasets generated for this study are available on request to the corresponding author.

## Ethics Statement

The studies involving human participants were reviewed and approved by the Comité Etico Científico Universidad de La Frontera. Written informed consent to participate in this study was provided by the participants’ legal guardian/next of kin.

## Author Contributions

JG-N created the research question, conducted the bibliographic search, methodological design, contributed to the analysis, results, and discussion. FP performed the data collection, contributed to the analysis, results, and discussion. IT-H contributed to the methodological design, performed the data analysis, and generated the results. KP-L conducted the bibliographic search, theoretical framework, and contributed to the discussion. JT-A conducted the bibliographic search, theoretical framework, and integrated results. All authors contributed to the article and approved the submitted version.

## Conflict of Interest

The authors declare that the research was conducted in the absence of any commercial or financial relationships that could be construed as a potential conflict of interest.
